# Supplementary material for: Redefining the multidimensional clinical phenotypes of frontotemporal lobar degeneration syndromes
Source: Brain. 2020 May;143(5):1555–71. doi: 10.1093/brain/awaa097 (PMC7241953; doi:10.1093/brain/awaa097)
Supplement: awaa097_Supplementary_Data [file awaa097_supplementary_data.pdf]

Clinical feature group (for PCA)	Symptom/Sign recorded at PIPPIN study assessment
Behavioural disinhibition/impulsivity	Socially inappropriate behaviour<br>Loss of manners or decorum<br>Impulsive rash careless action
Apathy	Apathy<br>Inertia
Loss of sympathy/empathy	Diminished response to other people's needs and feelings<br>Diminished social interest, interrelatedness or personal warmth
Stereotyped/compulsive behaviours	Simple Repetitive movements<br>Complex compulsive or ritualistic behaviour<br>Stereotypy of speech
Hyperorality/dietary change	Altered food preferences<br>Binge eating, increased ETOH or Cigarettes<br>Oral exploration of inedible objects
Executive dysfunction	Deficits in exec tasks<br>Relative sparing of episodic memory<br>Relative sparing of visuospatial skills
Asymmetrical parkinsonism	Asymmetric limb rigidity<br>Asymmetric limb akinesia
Asymmetrical dystonia	Asymmetric limb dystonia
Asymmetrical myoclonus	Asymmetric limb myoclonus
Symmetrical parkinsonism	Symmetric limb rigidity<br>Symmetric limb akinesia
Symmetrical dystonia	Symmetric limb dystonia
Symmetrical myoclonus	Symmetric limb myoclonus
Axial rigidity	Proximal rigidity more than peripheral<br>Axial rigidity or akinesia<br>Abnormal neck posture
Poor or absent response to L-dopa	Poor or absent response to L-dopa
Orobuccal apraxia	Orobuccal apraxia
Limb apraxia	Limb apraxia
Cortical sensory deficit	Cortical sensory deficit
Alien Limb	Alien Limb
Visuospatial deficits	Visuospatial deficits
Postural instability	Postural instability with tendency to fall<br>Frequent unprovoked falls within 3 years<br>Tendency to fall on the pull test<br>>2 steps backwards on pull test
Supranuclear gaze palsy	Vertical supranuclear gaze palsy<br>Decreased velocity of vertical saccades
Agrammatic/apraxic speech	Agrammatism in language production<br>Effortful halting speech with inconsistent sound errors (AoS)<br>Impaired comprehension of syntactically complex sentences
Impaired semantics	Impaired confrontation naming<br>Impaired single word comprehension<br>Impaired object knowledge<br>Surface dyslexia or dysgraphia
Logopenic speech	Impaired single word retrieval in spontaneous speech and naming<br>Impaired sentence repetition<br>Phonological errors in spontaneous speech
Motor neuron disease	Clinical signs of motor neuron disease

Section 1: The PIPPIN study diagnostic review form. Clinical features were grouped according to the first column prior to PCA

	Syndrome Dimension 1	Syndrome Dimension 2	Syndrome Dimension 3	Syndrome Dimension 4	Syndrome Dimension 5	Syndrome Dimension 6
Disinhibition	**0.7399**	0.0774	-0.0790	-0.1423	-0.1576	0.1102
Apathy	**0.5486**	0.0763	**0.4276**	0.1221	-0.1919	0.1450
Loss of sympathy or empathy	**0.7022**	0.1278	0.0044	-0.0981	-0.1278	0.0205
Stereotyped/compulsive behaviour	**0.5789**	0.2890	-0.3103	-0.1798	-0.0536	0.2444
Hyperorality/dietary change	**0.6234**	0.0459	-0.2198	-0.1270	-0.0932	0.0705
Executive dysfunction	**0.5458**	0.1440	0.1176	-0.0176	-0.0476	0.3155
CBI - Abnormal behaviour	**0.7497**	0.1251	-0.0348	-0.0545	-0.0898	-0.3164
CBI - Mood	**0.5485**	0.1013	0.0039	0.1709	-0.0077	**-0.5067**
CBI - Eating habits	**0.7647**	0.0394	-0.0486	-0.0500	-0.0074	-0.2188
CBI - Sleep	**0.4569**	-0.0259	0.2813	0.1776	-0.0488	**-0.4043**
CBI - Motor behaviour	**0.7056**	-0.0161	-0.1997	-0.1174	0.0726	-0.2513
CBI - Motivation	**0.7075**	0.2981	0.1511	0.0500	-0.1157	-0.1488
ACER - Attention/orientation	-0.1510	**-0.9002**	0.0922	0.0463	-0.0248	0.0724
ACER - Memory	-0.1124	**-0.8258**	0.3410	0.1746	-0.0440	0.0770
ACER - Fluency	-0.1784	**-0.7576**	0.1183	0.1772	-0.0744	-0.1227
ACER - Language	-0.0805	**-0.8460**	0.3405	0.1337	0.0314	0.0238
ACER - Visuospatial	-0.0532	**-0.8299**	-0.1642	-0.1818	-0.0460	0.1518
CBI - Memory	**0.4864**	**0.5544**	-0.2152	-0.0392	0.0199	-0.3158
CBI - Everyday skills	**0.4086**	**0.5214**	0.3309	0.3198	0.0727	-0.1291
Symmetrical parkinsonism	0.0127	-0.0415	**0.7676**	-0.3673	0.0655	-0.0362
Axial rigidity	-0.0156	-0.1158	**0.8077**	0.0425	-0.0231	0.0669
Poor levodopa responsiveness	-0.1307	-0.1057	**0.6757**	0.0873	-0.0629	-0.0536
Postural instability	-0.0504	-0.1069	**0.7719**	0.1690	-0.0640	-0.0241
Supranuclear gaze palsy	-0.0938	-0.1144	**0.8132**	0.1045	-0.0473	0.0526
CBI - Self care	0.3459	0.3996	**0.4524**	0.3626	-0.0628	-0.0775
Impaired semantics	0.1510	0.3067	**-0.5187**	-0.2377	0.0353	0.1970
Asymmetrical parkinsonism	-0.0652	-0.0843	0.0700	**0.8343**	-0.0627	0.0202
Asymmetrical dystonia	0.0282	-0.0673	0.0899	**0.8300**	-0.0550	0.1061
Asymmetrical myoclonus	-0.0340	-0.0148	-0.0493	**0.6830**	0.1012	0.0621
Limb apraxia	-0.1292	-0.0590	0.1252	**0.5274**	**0.5056**	-0.0233
Cortical sensory loss	-0.1462	-0.0201	0.0584	**0.5635**	0.2569	-0.2505
Alien limb syndrome	-0.0363	-0.0066	0.0504	**0.5423**	0.0749	-0.1367
Symmetrical myoclonus	-0.0658	-0.0350	0.0044	-0.0093	**0.5132**	-0.3228
Agrammatic, apraxic speech	-0.1224	0.1231	-0.0369	0.1137	**0.7667**	0.2703
Logopenic speech	-0.0659	0.0268	-0.1180	-0.0461	**0.7752**	0.0415
CBI - Beliefs	0.1830	0.2358	0.0220	-0.0019	0.0093	**-0.5919**
Symmetrical dystonia	0.1134	0.1176	0.3325	-0.1741	0.2010	0.0008
Orobuccal apraxia	-0.1225	-0.0170	-0.0012	0.2822	0.3727	-0.0267
Visuospatial deficits	-0.1863	0.1862	-0.0106	0.2386	0.3650	-0.2559
Motor neuron disease	0.2615	-0.1304	-0.1584	-0.0617	-0.1683	0.0556

Section 2: Varimax-rotated component matrix from principal component analysis. ACER: Addenbrooke's Cognitive Examination – Revised. CBI: Cambridge Behavioural Inventory. Positive loadings indicate worse performance or

presence of symptoms, except for ACER where negative loadings indicate worse performance. Factor loadings above 0.4 or below -0.4 shown in bold.

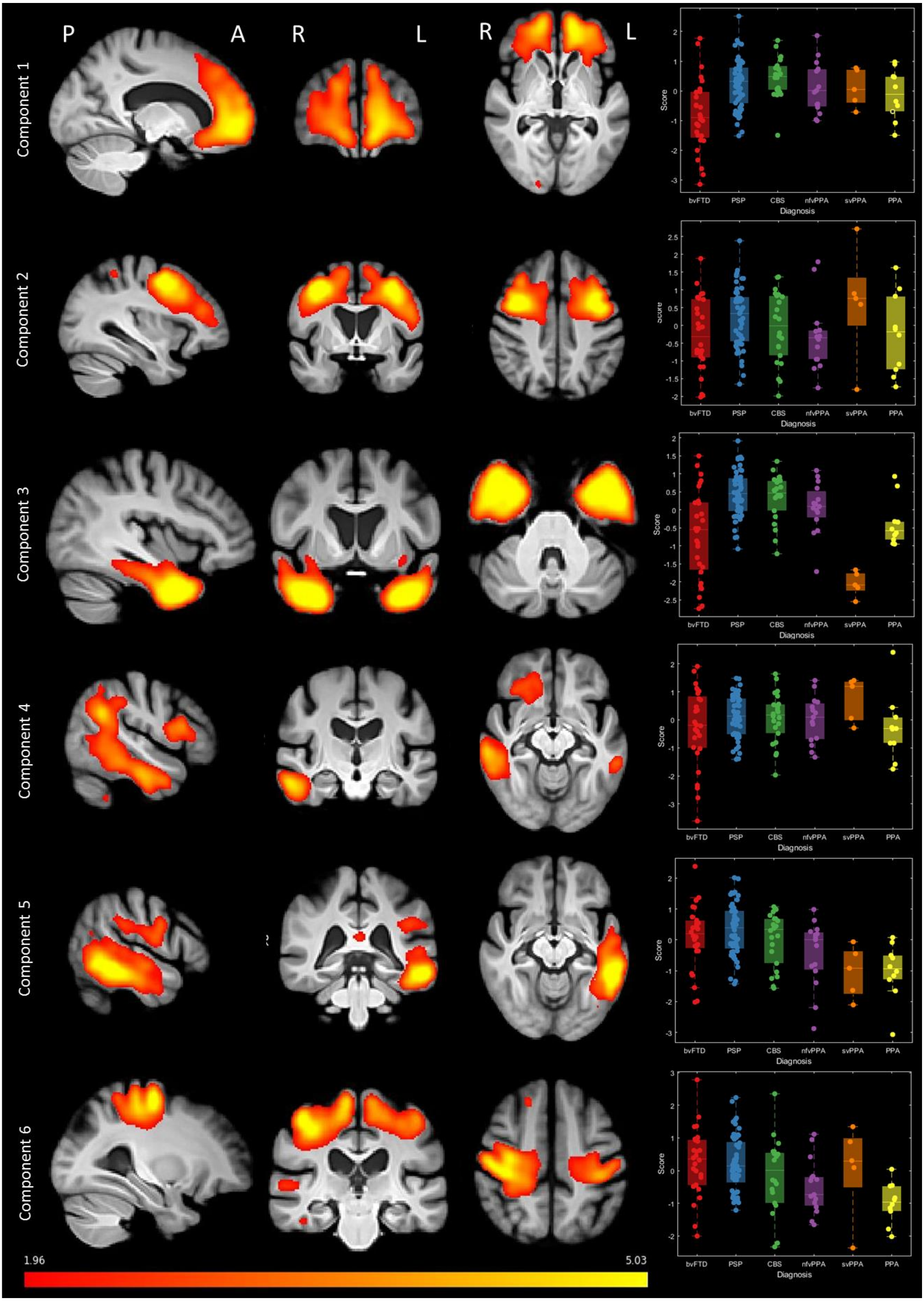
P
A
R
L
R
L
Component 1
Component 2
Component 3
Component 4
Component 5
Component 6
Score
Diagnosis
bvFTD
PSP
CBS
nfvPPA
svPPA
PPA
1.96
5.03

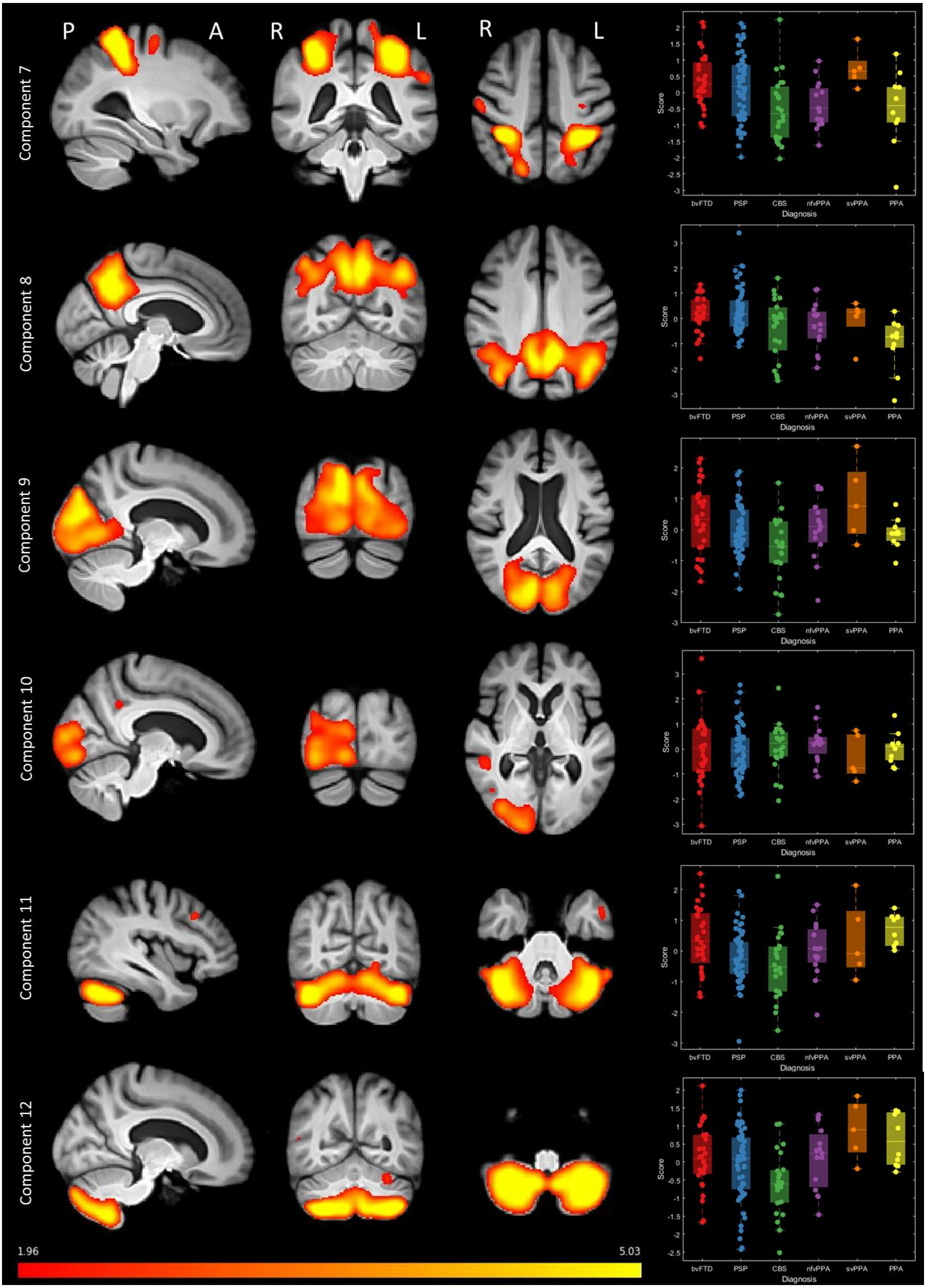
P
A
R
L
R
L
Component 7
Component 8
Component 9
Component 10
Component 11
Component 12
1.96
5.03
Score
Diagnosis
bvFTD
PSP
CBS
nfvPPA
svPPA
PPA

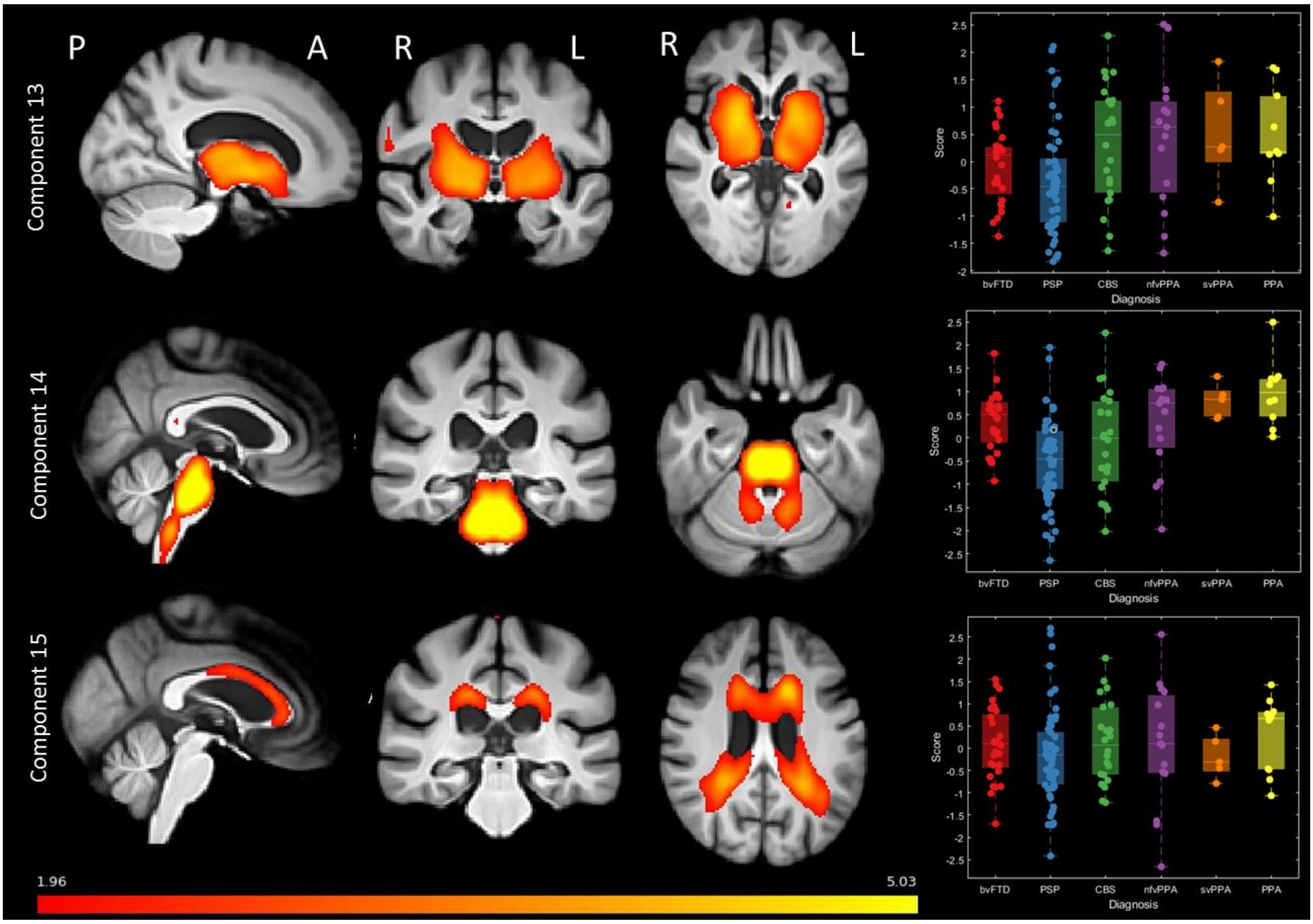

Section 3: Source based morphometry (based on independent component analysis) of combined grey and white matter. A total of 15 components were selected, each representing a region of independently covarying grey and white matter atrophy. Images are standardised group spatial maps for each component, superimposed on an average of all brain images. The scatter-box plots show the standardised subject loading coefficients, grouped by FTLD syndrome subtype. The variance (of atrophy across all participants) explained by component is shown below.

Component number	**1**	**2**	**3**	**4**	**5**	**6**	**7**	**8**	**9**	**10**	**11**	**12**	**13**	**14**	**15**
Variance explained (%)	8.7	8.9	3	6.4	7.6	2.7	0.5	4.9	6.5	0.2	3.9	3.6	17	3.4	0.5

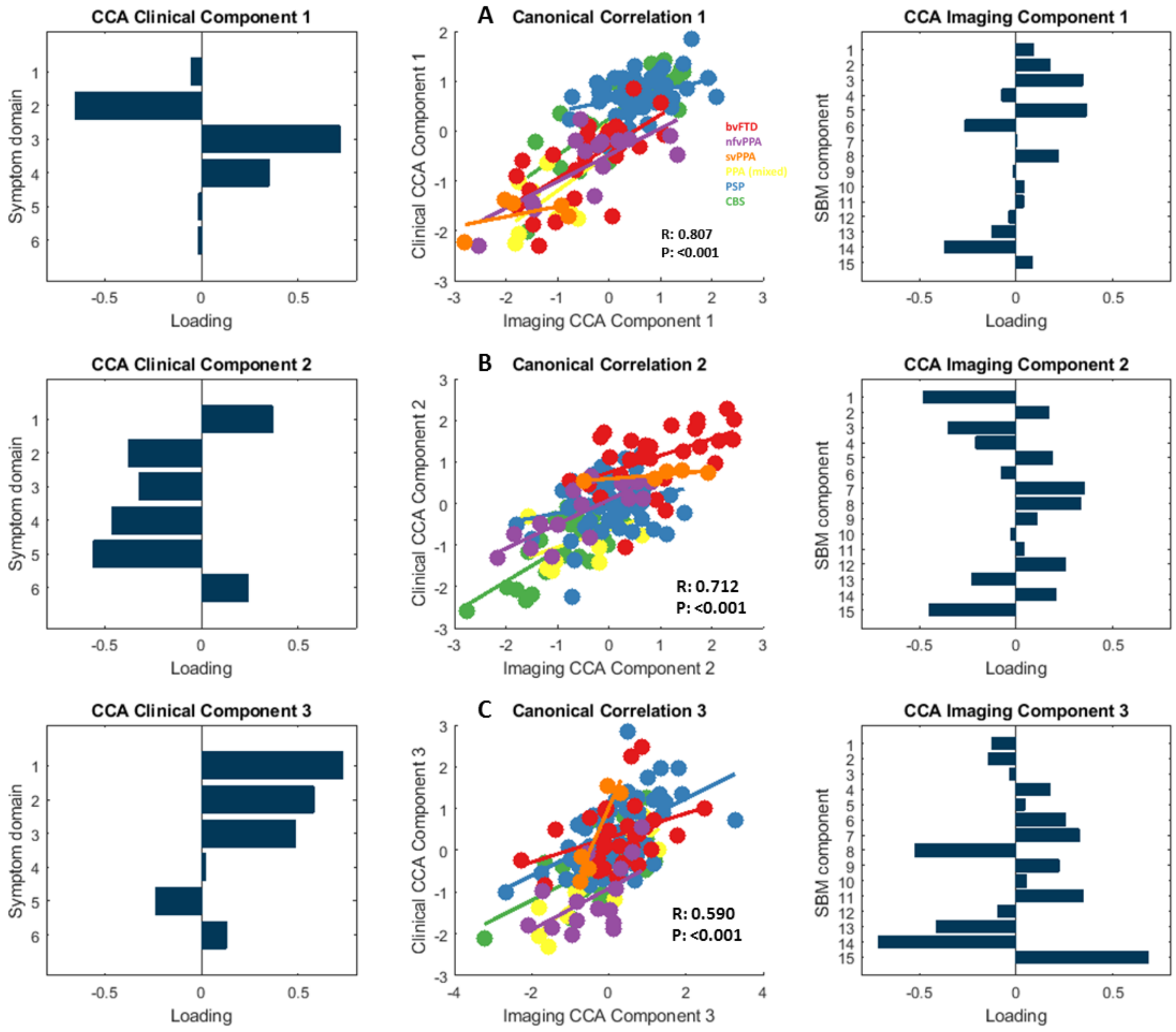

	bvFTD	svPPA	nfvPPA	PPA	PSP	CBS
**1st Canonical Correlation (R)**	0.6955	0.4957	0.7493	0.6092	0.3285	0.8077
**p value (FDR)**	0.0002	0.3957	0.0034	0.0791	0.0294	0.0001
**2nd Canonical Correlation (R)**	0.4871	0.7866	0.7946	0.4576	0.2374	0.7000
**p value (FDR)**	0.0171	0.1287	0.0014	0.1944	0.1043	0.0013
**3rd Canonical Correlation (R)**	0.3691	0.9140	0.5418	0.8729	0.5433	0.6169
**p value (FDR)**	0.0737	0.0489	0.0554	0.0029	0.0002	0.0050

Section 4: Canonical correlation analysis of clinical (syndrome dimensions from PCA) and imaging (atrophy components from SBM) components. The left column shows the loading plots from each syndrome domain onto the first, second and third clinical canonical components respectively. The right column shows the atrophy component loadings onto the imaging canonical components. The middle plots show the correlations between the clinical and imaging components. The table shows the correlations for each subgroup. P values are false discovery rate corrected for multiple comparisons and $p<0.05$ highlighted in blue.

Neuropathological validation of the study cohort

The forty-nine patients with a neuropathological diagnosis had a similar age ($t_{(309)}$=-0.7 p=0.48) and gender (X^2=0.52 p=0.47) distribution compared to whole study population but on average were assessed later in their illness ($t_{(309)}$=2.67, p=0.008). Twenty-one patients had a full dataset of clinical phenotype, neuropathological diagnosis and imaging. Of the 49 cases with *post-mortem* data, all patients with a clinical diagnosis of PSP had PSP pathology (n=14). Most patients with svPPA had FTLD-TDP-43 Type C (n=3) but one had Pick's disease. bvFTD was associated with FTLD-tau (Picks' n=1, PSP n=1) or TDP43 (n=5). Three patients with bvFTD had concurrent signs of motor neurone disease during the course of their illness, all with TDP43 pathology. Patients with CBS (n=19) had mainly CBD (n=6), Alzheimer's disease (n=8), multiple system atrophy pathology (n=2, despite normal autonomic function tests), FTLD-TDP43 (n=1) or PSP pathology (n=1). One CBS patient had micrometastatic renal cell carcinoma and paraneoplastic cerebellar degeneration. This patient had been treated for renal cell carcinoma but had no evidence of metastatic disease for over 3 years until a lung metastasis was diagnosed shortly before he died. He had no detectable serum autoimmune or paraneoplastic antibodies during the investigation of his corticobasal syndrome, and ataxia was not a prominent clinical feature despite the cerebellar degeneration. Of the four patients with nfvPPA, three had FTLD-tau and one had Alzheimer's disease. The one patient with lvPPA had Alzheimer's Disease pathology.

The number of patients with a neuropathological diagnosis was too small to test the classification accuracy of the syndrome dimensions or imaging components. This may be possible in future, as more patients in the study cohort undergo neuropathological assessment. We have included descriptive plots of the distribution of neuropathology in the syndrome dimensions, imaging components and canonical correlations. Any conclusions are tentative, due to the low numbers. Further research with larger sample size and cross validated classifier models is required to test the predictive accuracy of the syndrome dimensions for pathology.

Two of the syndrome dimensions have good clinicopathological associations (Section 5). Positive scores on syndrome dimension three was associated with FTLD-tau/PSP pathology. The majority of these patients had a clinical diagnosis of PSP, but one patient with bvFTD and a high score on syndrome domain 3 had PSP pathology. Interestingly, some patients with a clinical diagnosis of CBS or nfvPPA had high scores on domain 3 but CBD pathology at *post-mortem*. This PSP-like clinical phenotype associated with CBD pathology is well recognised (Alexander et al. 2013). All patients with Alzheimer's disease pathology had positive scores on syndrome dimension 5.

Volume loss in the brainstem was associated with FTLD-tau/PSP pathology. Basal ganglia atrophy (imaging component 13 in appendix 6) was associated with FTLD-TDP43 or FTLD-tau but not AD. No other imaging components were associated with specific neuropathology (Section 6). For example, all FTLD pathological subtypes were associated with volume loss in the frontal lobe (components 1 and 2). Parietal atrophy, reflected by low scores on imaging component 8, is typically associated with Alzheimer's disease pathology. Both cases of Alzheimer's disease had low scores, but so did some cases with FTLD-tau and FTLD-TDP43. Basal ganglia atrophy (imaging component 13 in appendix 6) was associated with FTLD-TDP43 and FTLD-tau but not AD.

Finally, we looked at the distribution of neuropathology within the three canonical correlation components, which show the multivariate relationships between clinical and imaging features (Section 7). In the first canonical correlation, positive scores were associated with FTLD-tau-PSP and negative scores with FTLD-TDP43 or Alzheimer's disease. Positive scores on the second canonical correlation were associated with FTLD-TDP43, and the three cases with AD pathology all had negative scores. The third canonical correlation did not clearly separate pathological subtypes.

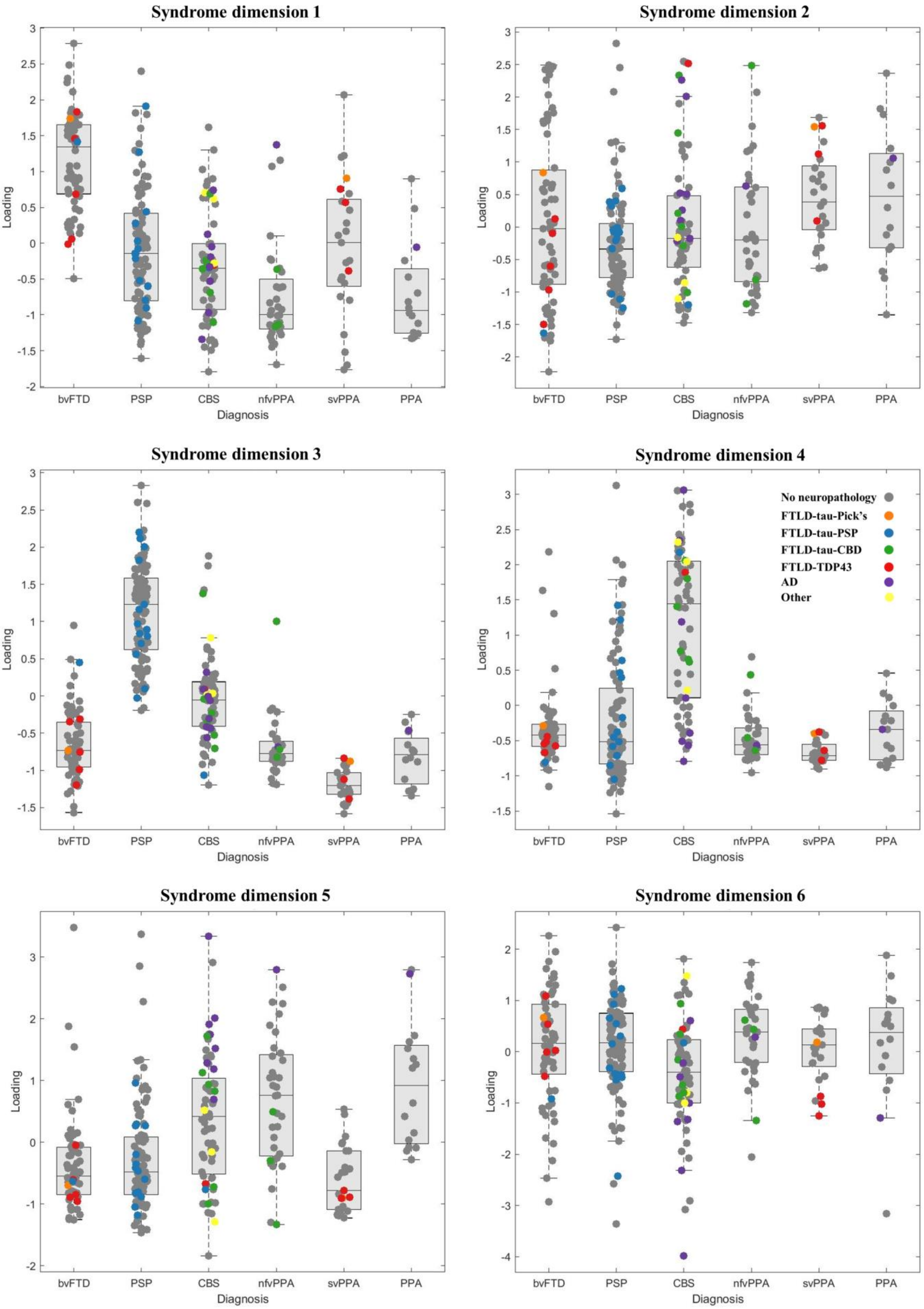
Syndrome dimension 1
Syndrome dimension 2
Syndrome dimension 3
Syndrome dimension 4
Syndrome dimension 5
Syndrome dimension 6
Loading
Diagnosis
bvFTD
PSP
CBS
nfvPPA
svPPA
PPA
No neuropathology
FTLD-tau-Pick's
FTLD-tau-PSP
FTLD-tau-CBD
FTLD-TDP43
AD
Other

Section 5: Boxplots of the participant scores on each syndrome dimension. Scatter plots are colour coded by neuropathological diagnosis (FTLD-tau-Pick's disease=orange, PSP=blue, CBD=green, TDP43=red, Alzheimer's disease (AD)=purple). Participants coloured grey were either still alive at the time of analysis or did not have a neuropathological diagnosis.

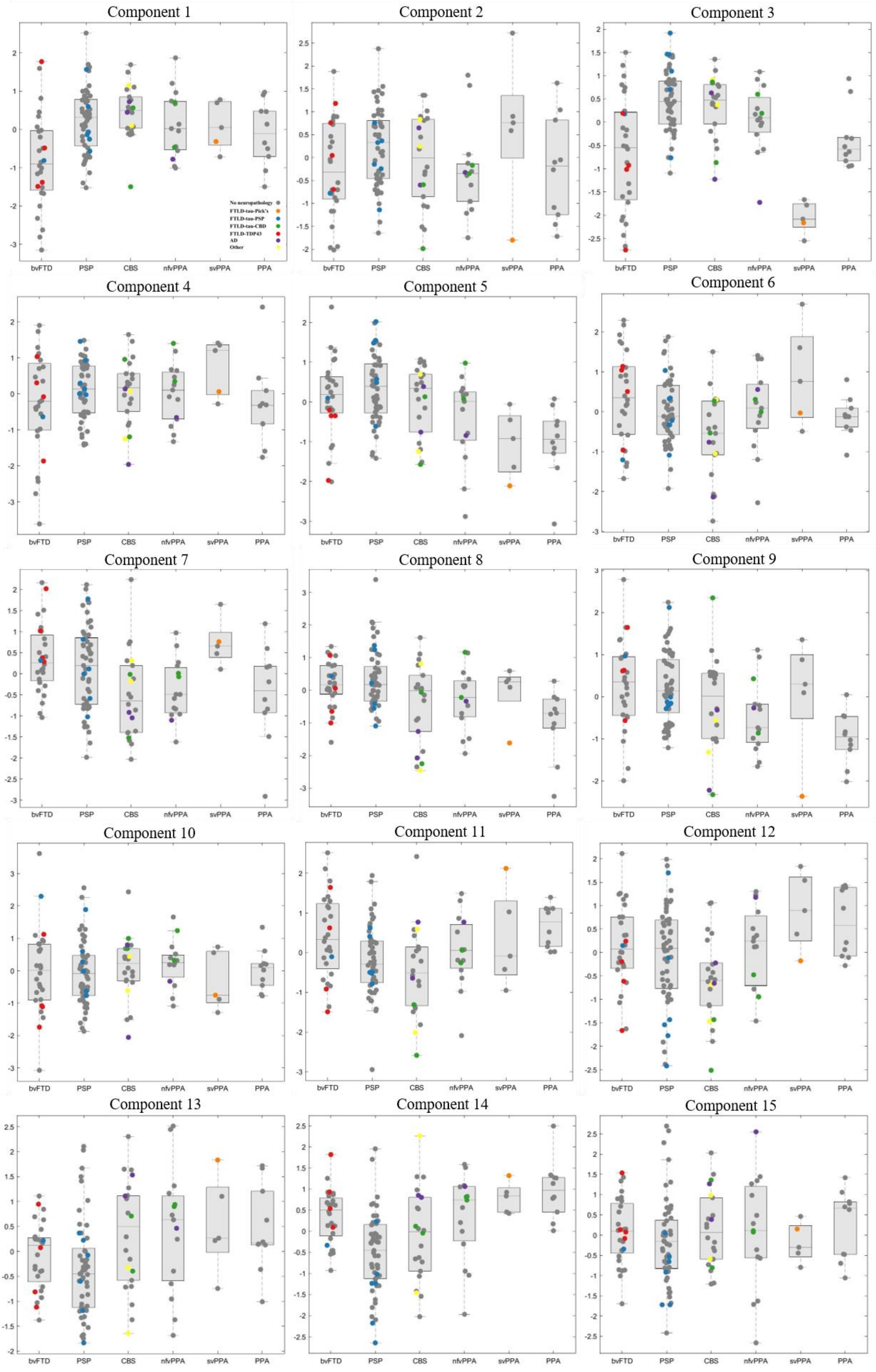

Section 6: Boxplots from Figure 4 of the participant scores on each source based morphometry imaging component dimensions. Scatter plots are colour coded by neuropathological diagnosis (FTLD-tau-Pick's disease=orange, PSP=blue, CBD=green, TDP43=red, AD, Alzheimer's disease=purple). Participants coloured grey were either still alive at the time of analysis or did not have a neuropathological diagnosis.

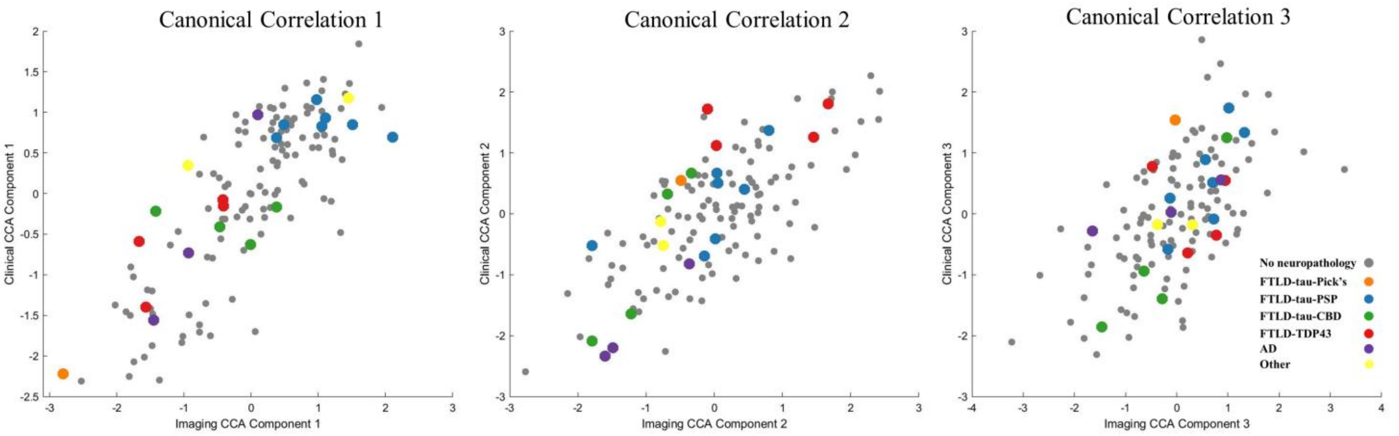

Section 7: Scatter plots of the canonical correlation analysis of clinical and imaging components. Points are colour coded by neuropathological diagnosis (FTLD-tau-Pick's disease=orange, PSP=blue, CBD=green, TDP43=red, Alzheimer's disease (AD)=purple). Participants coloured grey were either still alive at the time of analysis or did not have a neuropathological diagnosis.
